# Integrating forest data and health facility surveys to optimise risk-based malaria surveillance in the Philippines

**DOI:** 10.3389/fpubh.2025.1699392

**Published:** 2025-12-18

**Authors:** Kimberly M. Fornace, Ralph A. Reyes, Maria Lourdes M. Macalinao, Jun-Sik Lim, Alison Paolo N. Bareng, Jennifer S. Luchavez, Julius Clemence R. Hafalla, Fe Esperanza J. Espino, Jason Matthiopoulos, Chris J. Drakeley

**Affiliations:** 1 Saw Swee Hock School of Public Health and National University Health System, National University of Singapore, Singapore, Singapore; 2 Centre for Nature-based Climate Solutions, National University of Singapore, Singapore, Singapore; 3 Faculty of Infectious and Tropical Diseases, London School of Hygiene and Tropical Medicine, London, United Kingdom; 4 School of Biodiversity, One Health and Veterinary Medicine, University of Glasgow, Glasgow, United Kingdom; 5 Department of Parasitology, Research Institute for Tropical Medicine, Manila, Philippines

**Keywords:** malaria, surveillance, land use change, spatial epidemiology, easy access groups

## Abstract

**Introduction:**

Malaria transmission is highly spatially heterogeneous. Within Southeast Asia, forested landscapes are associated both with increased malaria transmission and reduced healthcare access. Identifying environments with malaria foci is a priority for control and elimination programmes.

**Methods:**

Here, we integrate health facility and environmental data to identify optimal surveillance approaches across a forested district in the Philippines. We conducted convenience surveys of health facility attendees utilising tablet-based applications to geolocate participant residences. Malaria infection was assessed using both routine (microscopy and rapid diagnostic test) and molecular methods. Integrating remote-sensing derived data, we assessed how fine-scale environmental factors influence the spatial distributions of malaria infections, diagnostic sensitivity and health-seeking behavior. We evaluated costs and probability of detecting malaria foci for multiple surveillance approaches using different diagnostic methods and target populations defined by landscape data.

**Results:**

We demonstrate that health facility-based surveys increase the probability of detecting malaria infections by increasing numbers of individuals screened and spatial coverage of surveillance systems. We additionally show sensitivity of routine malaria diagnostics varies spatially, with the decreased sensitivity in forests. By targeting diagnostic methods to high-risk environments, we developed a model approach for how to use landscape data within disease surveillance systems. Risk-based surveillance incorporating forest data is highly cost-effective and increases the probability of detecting malaria foci over three-fold compared to routine surveillance.

**Discussion:**

Together, this illustrates the essential role of environmental data in designing risk-based surveillance to provide an operationally feasible and cost-effective method to characterise malaria transmission.

## Background

Vector-borne disease transmission is highly variable spatially, driven by the geographical distribution of human populations, insect vectors and their environments. Within Southeast Asia, malaria risks are strongly associated with disturbed forest edges where *Anopheles* vector habitats overlap with rural settlements and populations with high occupational risks (1). Described as “frontier malaria,” factors associated with increased malaria risks, such as proximity to forest edges or deforestation, may be further amplified by limited healthcare and control programme coverage in remote areas or informal frontier settlements (2). As countries move towards malaria elimination, these forested areas remain some of the last foci of malaria transmission (1).

Surveillance systems aim to identify these high-risk locations to effectively plan, implement and evaluate control measures (3). For surveillance systems relying on reported malaria case data (passive surveillance), understanding spatial distributions of risk is challenged by underreporting due to health-seeking behavior or asymptomatic infections present in the community. Increasing evidence suggests the proportion of asymptomatic malaria infections not detectable by standard diagnostics increases in low transmission settings, resulting in large numbers of infections not detected by passive methods reliant on patients reporting to clinics (4, 5). These asymptomatic infections are commonly seen in older age groups, with potentially different risk factors and spatial distributions from clinical malaria cases (6, 7). Population-based community surveys (active surveillance) remain the gold standard for assessing spatial patterns of infection; however, these sampling approaches are highly resource-intensive, need to be frequently repeated and may require very large sample sizes in low transmission settings. Alternatively, more operationally feasible surveys of easy access groups (convenience sampling), such as health facility attendees or school children, are used to increase probability of detecting infections within the community (8).

Both passive and convenience surveillance approaches are inherently biased due to imperfect detection and spatially biased observation processes. Bayesian latent process modelling approaches have been used to estimate the probability of presence within a geographic location while accounting for possible non-detection (9). Models can partition observation processes determining detection probability and biological processes determining probability of presence, each associated with potentially overlapping spatial and environmental covariates. This makes the simple assumption that a disease cannot be detected if it is not present; however, if present, the disease may or may not be detected during sampling. In addition to allowing estimation of true distribution of disease as a latent variable, this method allows quantification of uncertainty in the observation process under different sampling scenarios (10).

**Figure 1:**
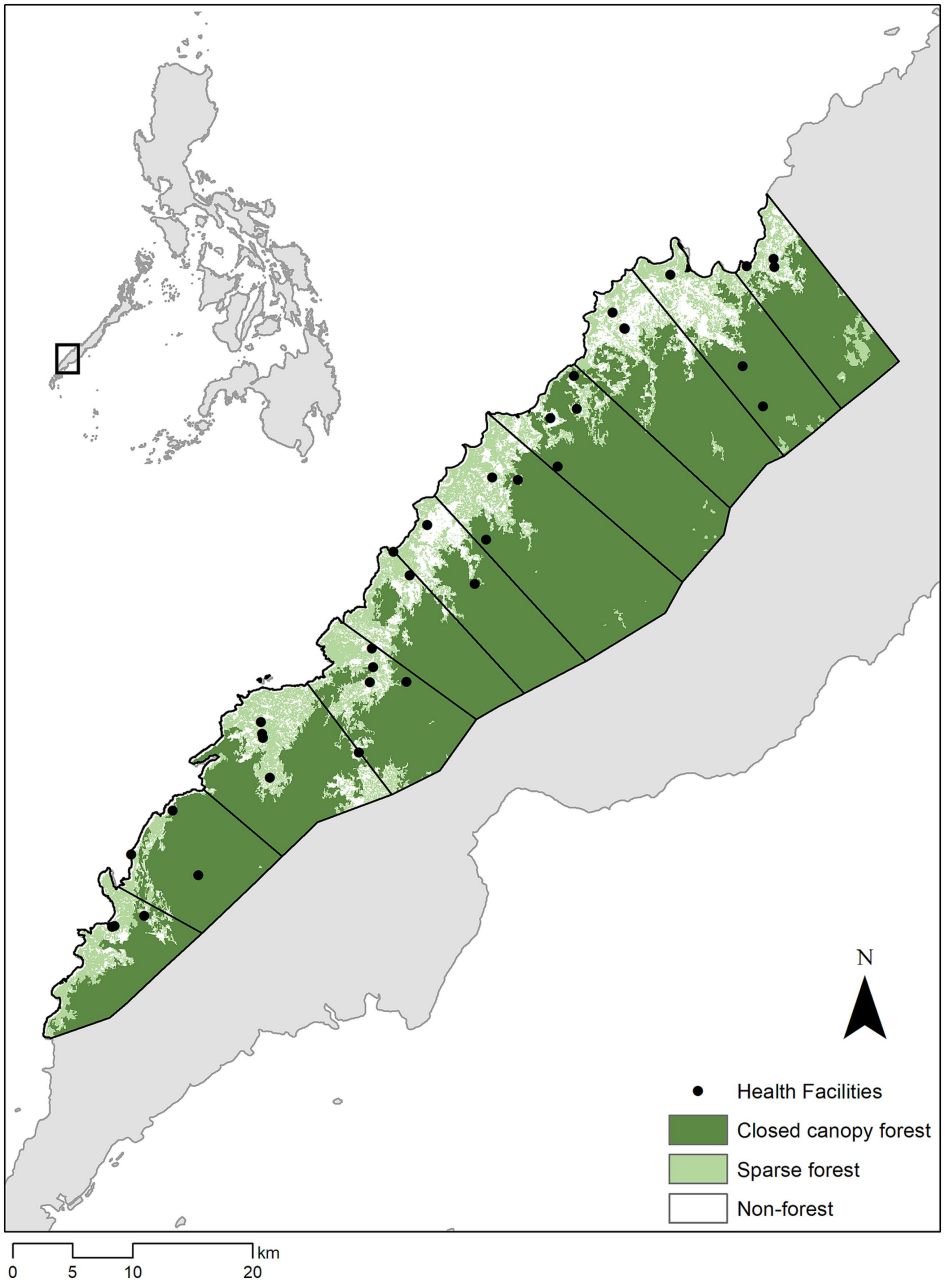
Study area and forest cover in Alos-2/Alos Science Project Earth Observation Research Center (Eorc) (24), higher elevation areas showed by shading.

Here, we combine these modelling approaches with health facility surveys of easy access groups and molecular diagnostics to estimate the underlying distribution of infections and optimise surveillance approaches for the forested municipality of Rizal, Palawan, The Philippines (Figure 1). The Philippines has made substantial progress towards malaria elimination, with all provinces declared malaria free except for Palawan (11). Within Palawan, malaria transmission occurs primarily in rural indigenous populations living near forest environments (12). Rizal municipality reports most malaria cases for the Philippines (13, 14) as well as the highest rates of deforestation in Palawan (15). Of the 33% decrease in forest cover in Rizal since 2000, the majority of deforestation occurred after 2015, largely driven by agricultural expansion and extractive industries (16). However, land cover data rarely informs the design of surveillance systems.

We aimed to develop cost-effective, environmentally targeted malaria surveillance systems to increase the probability of detecting locations with malaria infections, assessing inclusion of different surveillance and diagnostic methods. Within the Philippines, identification of areas with malaria infections triggers reactive case detection. As subsequent community-based activities provide more robust quantification of malaria burdens, we assessed the capacity of surveillance approaches to detect locations with infections rather than malaria prevalence or incidence. By estimating spatially explicit probabilities of detection, we illustrate how environmental data can be used to develop operationally feasible risk-based surveillance systems. Integrating forest data into targeted surveillance approaches, we develop cost-effective risk-based surveillance methods to identify locations with high probabilities of infection.

## Methods

### Health facility-based surveys

We conducted monthly rolling cross-sectional surveys at 27 health facilities across Rizal, Palawan over a one-year period between June 2016 – June 2017 (12). This municipality has a primarily rural population of approximately 50,000, including a high proportion of indigenous groups. The predominant occupational activities are agricultural, mostly consisting of small-scale swidden farming. Despite rapid deforestation, large areas of primary forest remain in high elevation areas (16). Rizal reports the highest malaria incidence within the Philippines and has an active malaria control programme and network of health facilities and community health workers. Rapid diagnostic tests (RDTs) and microscopy are routinely used to diagnose malaria within these clinics while blood spots for molecular analysis can be collected but only analysed at central laboratories.

Health facilities surveyed included one Regional Health Unit (RHU), the central health unit for Rizal, as well as 9 Barangay Health Stations (BHS) and 17 RDT centres based in community health worker households (Supplementary Figure S1). For one week every month, we surveyed all individuals presenting to the health facility, regardless of symptoms or patient status (e.g., including patients seeking treatment and accompanying companions). For consenting individuals, malaria status was assessed using microscopy or RDTs, with finger-prick blood samples collected on Whatman 3MM filter paper for subsequent analysis using molecular methods (12). We classified individuals as malaria positive if any *Plasmodium* species were detected by any diagnostics. Basic demographic information and self-reported household locations were identified using offline satellite maps on GeoODK on Android tablets (Supplementary Figure S2) (17).

### Definition of surveillance systems

To evaluate possible surveillance approaches, we initially defined two surveillance methods: (1) Standard passive case detection (PCD), in which all febrile cases were screened for malaria using routine diagnostics (RDT or microscopy) as per national guidelines and (2) Enhanced surveillance, in which all febrile cases and health facility attendees were screened with both routine and molecular diagnostics. We compared the ability of surveillance methods to detect locations with malaria infections, defined as at least one *Plasmodium* sp. positive individual confirmed using any diagnostic method. Subpatent malaria was defined as *Plasmodium* sp. infections detectable by molecular methods but not RDT or microscopy.

To identify the locations of all households in Rizal, we extracted information on household structure locations from the Facebook High Resolution Settlement Layer, a 30 m resolution satellite-based remote sensing derived dataset on all inhabited structures (18). This dataset was combined with all reported households identified by survey participants and geolocated households from the 2015 Philippine census (19). Datasets were resampled to 50 m resolution and duplicate locations removed to generate a complete dataset of the spatial extent of households in Rizal. To estimate detection probabilities, we classified locations as included by a surveillance method if at least one person in that location was tested for malaria.

### Spatial and environmental covariates

Plausible covariates used to model detection or infection probabilities were assembled (Supplementary Table S1). These included forest, climatic and topographic factors associated with *Anopheles* mosquito habitats within this region as well as factors associated with healthcare access such as roads and population density. Handheld GPS devices (Garmin, United States) were used to record locations of all sampled clinics and roads within the region. Travel time to the nearest sampled clinic was calculated as accumulated cost from friction surfaces (20). Additional covariates included population density (21), Euclidean distance from roads and bioclimatic variables (22). Elevation and topographic measures, including topographic wetness index (TWI), upslope area and aspect, were calculated from the ASTER Global Digital Elevation Model (23). Forest cover was classified as over 50% canopy cover and Euclidean distance was calculated to the forest edge, recent deforestation within the past year and cumulative historical deforestation within the previous five years (16). We additionally included closed canopy forest, defined as canopy cover over 90% with a minimum area of 0.5 ha (24). Covariates were extracted for all household locations and Pearson’s correlation coefficient was used to assess multicollinearity.

### Bayesian modelling

We initially fit separate models for detection (whether a household was sampled) and infection (malaria presence or absence) using datasets representing PCD and enhanced surveillance methods (Supplementary Figure S3) (25, 26). Nonspatial binomial generalised linear models were fit separately for detection and infection for each surveillance dataset using a backwards stepwise model selection approach with a five-point threshold for improvement in deviance information criteria (DIC) to minimise overfitting (27). Residual spatial autocorrelation was assessed using Moran’s I and predictive performance assessed by area under the receiver operating curve (AUC). Weakly informative priors of Normal (0,100) were used for all intercepts and coefficients. All models were implemented in Integrated Nested Laplace Approximation (INLA), with 10,000 samples generated from the approximated posterior distribution (28).

We modelled distributions of infections under each surveillance method *k* separately using occupancy models in which the probability of detecting an infection (*y_i,k_*) in location *i* is dependent on the probability of detection (*p_i,k_*) and presence of infection (*ω_i_*) (9), modelled as:


$$y_{i.k}\sim \{\begin{array}{cc} 0, & \omega_{i}=0\\ \text{Bernoulli}(p_{i.k}), & \omega_{i}=1 \end{array}$$

Where the linear predictor determining the probability of detection for surveillance method *k* is modelled as:


$$\text{logit}(p_{i,k})=\alpha_{0}+X_{i,k}\alpha +u_{i.k}$$

Where $\alpha_{0}$ represents the intercept, $X_{i,k}\alpha$ represents a vector of covariate effects with α representing the coefficients for covariate data from location *i* ($X_{i,k}$) and $u_{i,k}$ is the spatial effect modelled as a Matern covariance function using the stochastic partial differential equations approach to represent the spatial process by Gaussian Markov random fields as implemented in INLA (28, 29). The process determining the true state of malaria presence *ω* is determined by the true probability of infection *ψ*:


$$\omega_{i}\sim \text{Bernoulli}(\psi_{i})$$

With the linear predictor for the Bernoulli model specified as:


$$\text{logit}(\psi_{i})=\beta_{0}+X_{i}\beta +\gamma_{i}+Zu_{i}$$

Where $\beta_{0}$ represents the intercept, $X_{i}\beta$ represents a vector of covariate effects and $\gamma_{i}$ represents the spatial effect, modelled as described above. As processes influencing probability of detection (healthcare access) additionally may impact infection, we include a shared spatial component $Zu_{i,k}$with scaling parameter *Z* (30).

To explore factors affecting the spatial distribution of patent malaria infections compared to all infections, we subset all malaria infected locations. For *J_j_* malaria infected individuals identified in each location, the number of patent infections observed (*m_i_*) is modelled as:


$$m_{i}\sim \text{Binomial}(J_{i},s_{i})$$

With the linear predictor determining the probability of patent infections (*s_i_*) modelled as:


$$\text{logit}(s_{i})=\kappa_{0}+X_{i}\kappa$$

Where $\kappa_{0}$ represents the intercept and $X_{i}\kappa$ represents a vector of covariate effects. As this model estimates the probability of patent infections (e.g., infections detected by RDT and/or microscopy) in all malaria infected individuals, this is equivalent to predicting the sensitivity of these diagnostics. Using data from all locations included in the study site, we then predicted a location specific sensitivity of routine diagnostics. Based on these results, we used environmental data to define an area with higher probabilities of malaria infections only detectable by molecular diagnostics.

### Evaluation of surveillance systems

We modelled the true probability of infection from the infection process model using data from all available diagnostics. We compared the performance of five surveillance methods, each with a different combination of survey and diagnostic methods (Table 1). First, we compared two survey methods: standard PCD and enhanced surveillance (including health facility attendees). Next, we combined these survey methods with different diagnostic methods, molecular or standard, generating 4 unique scenarios. Finally, we developed a risk-based approach using molecular diagnostics and health facility attendee surveys only in an area defined using forest data. Then, for each of the five surveillance approaches with different survey and diagnostic methods, we estimated the number of infected locations not detected as:


$$\sum_{i=1}^{i}\omega_{i}(1-p_{i,k})+\omega_{i}p_{i.k}(1-s_{i})$$

**Table 1:** Surveillance methods assessed.

	Survey method		Diagnostic method		Total cost (USD)
	Passive case detection	Health facility surveys	Routine (RDT/microscopy)	Molecular	
1: Standard PCD	X		X		-
2: Enhanced surveillance	X	X	X	X	193,547.70
3: PCD + molecular	X		X	X	56,654.40
4: Health facility surveys + routine	X	X	X		22,844.50
5: Risk-based surveys + diagnostics	X	Risk zone only	X	Risk zone only	97,764.67

Where *p_i_* is the probability of detection using different survey methods and *s_i_* represents diagnostic sensitivity for location *i*, with PCR considered the gold standard. We estimated the sensitivity of different combinations of survey and diagnostic methods. We additionally defined a risk-based surveillance approach targeting health facility surveys and molecular diagnostics to areas defined by proximity to closed canopy forests.

To evaluate the cost effectiveness of surveillance approaches, we estimated the additional costs to health systems of including different survey and diagnostic methods. Costs were calculated in Philippine pesos (PHP) converted into US dollars using the 2018 conversion rate (1 PHP = 0.02 USD). This excluded capital costs and costs already covered by existing health systems, routine diagnostics for febrile patients. Health facility survey costs included additional payments to personnel, training, equipment, sample collection and diagnostic costs for non-febrile participants. Molecular diagnostic costs included PCR and DNA extraction completed using Chelex with 10% of samples verified using a commercial Qiagen kit (12). As this study aimed to identify locations of infections, we evaluated costs per location of malaria infections detected. All analysis was completed in R statistical programming language (v3.6), with maps visualised in R or ArcGIS (ESRI, Redlands, United States). Data is available on reasonable request with approval from relevant ethics committees. All R code is available at: https://github.com/kfornace

## Results

Between June 2016–June 2017, 5,767 individuals were enrolled in this study, including 1,914 (33.2%) febrile patients (12). From all participants, 801 (13.9%) were malaria positive and 498 (8.6%) had patent malaria infections. *P. falciparum* was the most common parasite identified, comprising 74.3% of patent malaria infections and 66.5% of subpatent malaria infections (12).

**Figure 2:**
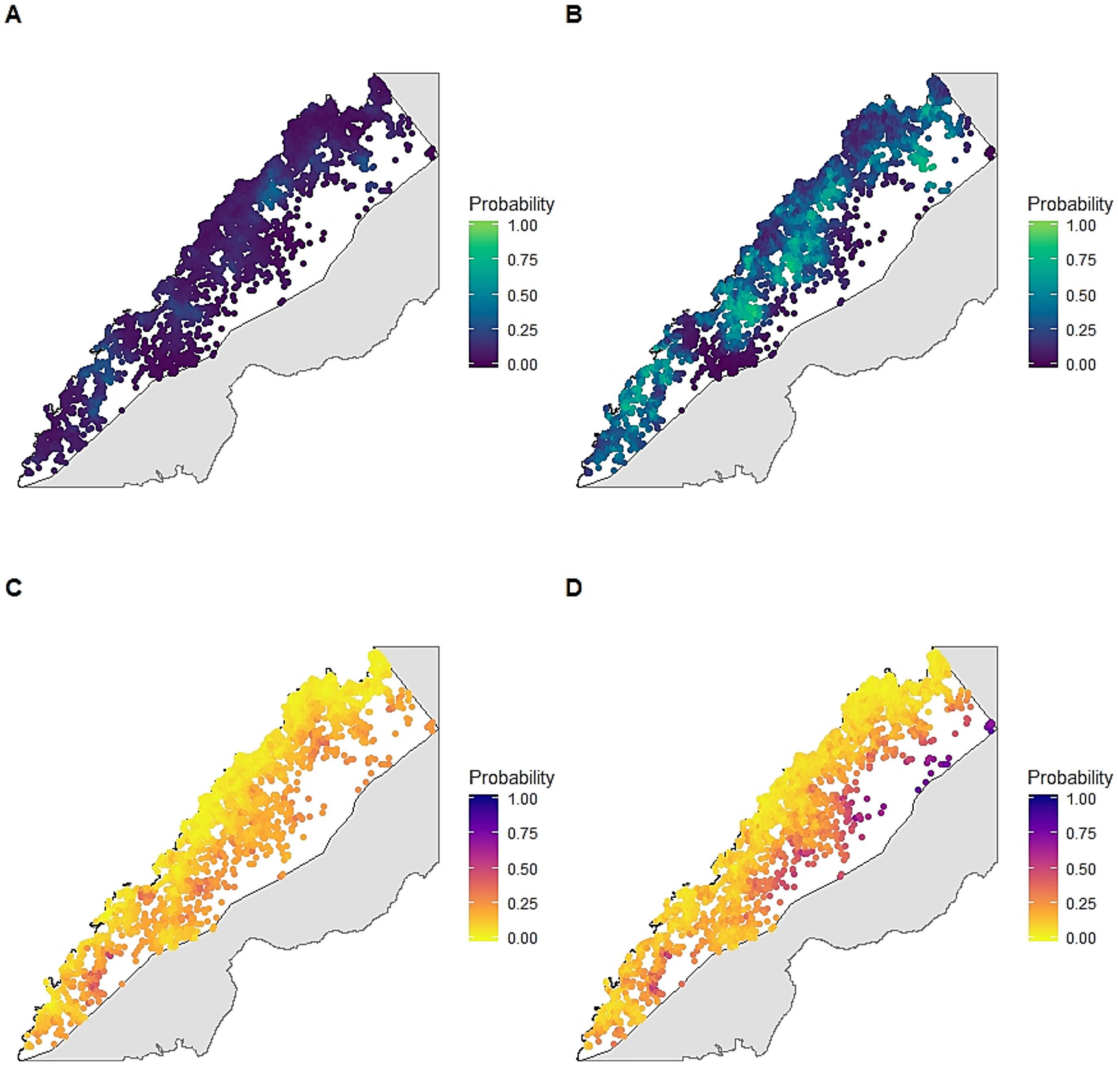
Posterior probability of infection under different sampling scenarios adjusted for detection probabilities: **(A)** detection probability using standard passive case detection; **(B)** detection probability using enhanced health facility-based surveys; **(C)** probability of infection estimated from passive case detection using routine diagnostics; **(D)** probability of infection estimated from enhanced health facility-based surveys using molecular diagnostics.

We geolocated all residence locations in Rizal (*n* = 7,313) from census and remote sensing-derived data (Supplementary Table S1). Individuals screened by PCD were identified from 698 unique locations while health facility surveys screened individuals from 2,201 locations (Supplementary Table S2). Malaria infections were detected at 352 locations using enhanced surveillance and 117 locations by PCD. Detection probabilities, the probability of screening at least one individual from a location during the study period, varied geographically, with travel time to the nearest health facility negatively associated with detection probabilities by both PCD and enhanced surveillance methods (Tables 2–4). Enhanced surveillance increased detection probabilities over three-fold compared to standard PCD (mean: 3.34, 95% Bayesian credible interval (BCI): 1.03–8.27) in addition to markedly increasing spatial coverage of surveillance, particularly near forested areas (Figures 2A,B).

**Table 2:** Spatial and environmental characteristics of all households in Rizal (data sources described in Supplementary Table S1).

Covariate	Mean	SD
Elevation (metres above sea level)	44.50	87.11
Topographic wetness index	7.24	1.91
Upslope area	7.98	0.94
Aspect	0.10	0.09
Population density	0.68	0.28
Distance to forest (m)	39.17	46.67
Distance to forest loss in the past year (m)	348.27	296.31
Distance to forest loss in past 5 years (m)	229.83	227.40
Distance to closed canopy forest (m)	1063.13	1236.11
Distance to roads (m)	867.40	1367.03
Travel time to clinic	20.54	44.34
Temperature annual range (°C)	9.42	0.25
Precipitation of wetness month (mm)	264.43	10.19

**Table 3:** Posterior estimates of fixed effects and spatial range [calculated as described by Lindgren and Rue (29)] for joint model of standard PCD.

	Mean	SD	95% BCI
Probability of detection			
Distance to roads	0.226	0.125	−0.020, 0.227
Travel time to clinic	−0.317	0.120	−0.561, −0.090
Distance to forest	−0.112	0.053	−0.217, −0.009
Spatial range (km)	8.140	2.500	4.319, 14.050
Probability of infection			
Distance from roads	0.094	0.101	−0.109, 0.287
Population density	−0.603	0.145	−0.894, −0.322
Precipitation of wetness month	0.212	0.107	−0.006, 0.420
Distance from closed canopy forest	−0.222	0.156	−0.537, 0.078
Spatial range (km)	1.752	1.096	0.493, 4.643
Scaling parameter for shared spatial effect	0.590	0.163	0.282, 0.921

** All covariates mean-centred and scaled.

**Table 4:** Posterior estimates of fixed effects and spatial range [calculated as described by Lindgren and Rue (29)] for joint model of enhanced surveillance.

	Mean	SD	95% BCI
Probability of detection			
Population density	−0.533	0.085	−0.701, −0.368
Travel time to clinic	−0.511	0.107	−0.729, −0.309
Aspect	0.094	0.039	0.017, 0.171
Spatial range (km)	15.804	6.130	7.212, 30.957
Probability of infection			
Distance from roads	0.285	0.071	0.145, 0.423
Upslope area	0.181	0.111	−0.038, 0.399
Topographic wetness index	−0.243	0.120	−0.481, −0.011
Temperature annual range	0.236	0.098	0.043, 0.428
Distance from closed canopy forest	−0.326	0.111	−0.548, −0.112
Spatial range (km)	0.897	0.232	0.532, 1.438
Scaling parameter for shared spatial effect	1.216	0.177	0.887, 1.179

** All covariates mean-centred and scaled.

Enhanced surveillance detected a much wider spatial distribution of malaria than PCD alone, identifying areas with infection not captured through routine surveillance (Figures 2C,D). We identified a range of different spatial and environmental risk factors for infections detected by different diagnostic methods; however, all infections were associated with proximity to closed canopy forests (Tables 2–4). For joint models incorporating detection and infection probabilities for both surveillance approaches, incorporating a shared spatial random effect between infection and detection probability improved model performance, suggesting a common spatial process driving healthcare access and disease risks (Supplementary Table S3).

To explore factors determining these differing infection distributions, we estimated the probability of patent malaria for all malaria infections identified. Malaria infected individuals were identified from 435 unique locations and over one third (37.8, 95% BCI: 34.5–41.3%) of infections could only be detected using molecular methods. Subpatent malaria was more common in forested areas, with the odds of patent malaria infections increasing 1.23 (95% BCI: 1.03–1.47) with every kilometre distant from closed canopy forests (Table 5). Using data from all residence locations, models were used to predict a spatially explicit probability of patent malaria, equivalent to the sensitivity of routine diagnostics (RDT and/or microscopy) (Supplementary Figure S4).

**Table 5:** Posterior estimates of fixed effects for malaria positive households detected by routine diagnostics (RDT and microscopy) compared to molecular methods.

	Mean	SD	95% BCI
Distance from closed canopy forest*	0.225	0.112	0.036, 0.476
Annual precipitation*	−0.318	0.121	−0.620, −0.145
Precipitation of wettest month*	0.256	0.085	0.091, 0.422
Population density^2^*	0.093	0.087	−0.078, 0.263

*Mean-centred and scaled.

**Figure 3:**
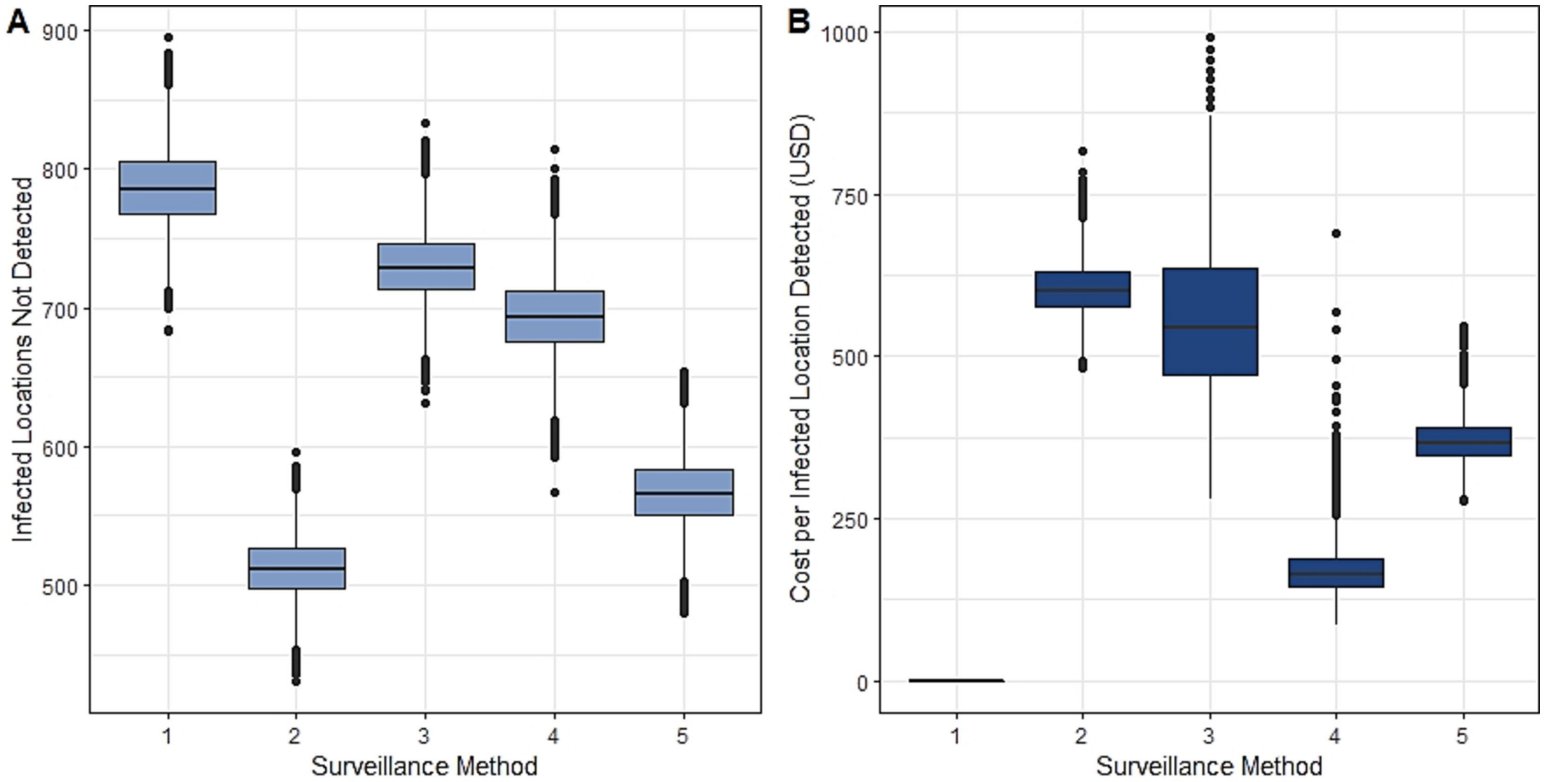
Evaluation of surveillance methods described in Table 1, including 1: PCD with routine diagnostics, 2: enhanced surveillance, 3: PCD with molecular diagnostics, 4: health facility surveys with routine diagnostics and 5: risk-based surveys, by **(A)** estimated numbers of locations with malaria infections not detected, and **(B)** estimated additional costs per location with malaria infections detected (relative to standard PCD).

Adjusting for detection bias, we inferred the true distribution of malaria infections as a latent variable from joint models fit using all available spatial and diagnostic information. We estimated 11.4% (95% BCI: 4.6—21.9%) of locations in Rizal had malaria infections during the study period. While no surveillance method perfectly identified the spatial distribution of infections, enhanced surveillance using molecular methods identified 38.7% (95% BCI: 33.6–43.8%) of locations with infections while PCD identified only 5.7% (95% BCI: 0.1–11.9%). Screening all febrile patients with molecular methods only slightly improved the probability of detecting infections while using routine diagnostics on all health facility attendees increased the number of infected locations detected slightly more (Figure 3A; Supplementary Figure S5). We additionally identified 247 locations with very low (<0.05% probability) of detection by any health facility-based surveillance.

Based on infection and detection probabilities, we additionally explored the used of environmentally stratified risk-based surveillance approaches based on distances from closed canopy forests (Supplementary Figure S6; Supplementary Table S4). We estimated the total cost per location with malaria infection identified relative to the baseline PCD approach (Figure 3B). As the cost per location of infection detected was most sensitive to inclusion of molecular diagnostics, we defined a risk-based surveillance approach using health facility surveys in all areas and only applying molecular diagnostics to individuals reporting residences within 100 m of closed canopy forest areas (Table 1; Supplementary Figure S7). This risk-based surveillance approach almost halved the cost of detecting a location of infection compared to enhanced surveillance, from USD 603.10 (95% BCI: 530.02–690.82) to USD 370.00 (95% BCI: 313.18–444.04) while detecting almost as many locations of infections.

## Discussion

Here, we demonstrate convenience approaches using health facility surveys markedly increases the spatial coverage of surveillance systems. Integrating these surveys with satellite-derived remote sensing data allows estimation of the underlying distribution of infections not captured by passive case detection. We additionally identify higher proportions of subpatent malaria infections in forested environments, limiting the utility of routine diagnostics in these settings. Integrating environmental data, we develop a cost-effective and operationally feasible risk-based surveillance approach and illustrate how landscape data can be incorporated into disease surveillance.

Despite extensive research linking forest proximity with malaria risks in Southeast Asia, landscape data are not routinely used to inform surveillance systems. Malaria control programmes typically conduct community-based active case detection in response to reported malaria cases (3); however, we show this may miss a substantial proportion of active malaria foci due to biases in health-seeking behavior and increased prevalence of subpatent malaria within higher transmission areas. Although mechanisms driving this relationship between forest cover and subpatent malaria are not known, patent malaria infections are more common in children in this area and settlements near forests may have different demographic compositions (e.g., logging and plantation camps) (12). Previous studies have also suggested a role for immunity in high transmission areas, with individuals repeatedly exposed to malaria having lower parasite densities (4). These subpatent infections can lead to infections in mosquitoes and may have a critical role in sustaining transmission in elimination settings (31–33).

Increasing availability of remote sensing data provides new opportunities to target surveillance activities. Surveillance systems for malaria and other low incidence diseases are challenged by the need to identify relatively rare events with shifting spatial patterns (34). Risk-based surveillance uses known risk factors to focus intensive surveillance activities on the populations where rare events are most likely to occur (35). In this study, we demonstrate that screening all individuals attending health facilities vastly increases the spatial coverage of surveillance. Additionally, risk-based surveillance using environmental data enables targeting diagnostics to identify malaria infections more cost-effectively. These methods were implemented using tablet-based applications to identify residence locations from offline satellite data (17). These tools can be further expanded to create accessible interfaces for local health workers to use environmental and spatial data and incorporate risk-based decision pathways on screening procedures and diagnostic tests based on household locations and travel history. This approach can also be easily modified to include multiple diseases with different underlying environmental risk factors or updated to include near real time environmental data, such as deforestation alerts (36). For example, rural health facility workers could use tablet-based applications or maps to determine whether to collect additional samples from an individual living in a high-risk area or an area where sub-patent malaria is more likely. While the exact goals and budget for this type of surveillance would need to be determined on a case specific basis, this can provide a method to optimise detection in resource limited settings. Despite advances in using meteorological data to forecast vector-borne diseases, landscape data is rarely used operationally and may provide more actionable information within rapidly changing environments.

Despite the utility of these methods, there were limitations to this study. As this study was designed to identify spatial locations of malaria infections within the sampling year, we did not explore temporal patterns of infection or health seeking behaviors. However, the modelling approach used is easily extendable to incorporate dynamic state-space models of changes in infection over time and seasonally varying meteorological data (37). As molecular assays are performed at a central laboratory, for this site, delays in receiving test results are likely to limit the utility of fine-scale temporal data within this setting. Additionally, while molecular approaches for malaria are not easily applied in rural settings, new diagnostics, such as lateral flow assays and serological tests, may facilitate point of contact testing in the future. While populations at risk were defined using multiple datasets, this is likely to have limited coverage of highly mobile indigenous populations not residing in permanent structures. Future work could explore the utility of satellite imagery to identify these populations, such as through monitoring of forest disturbance or modelling movement patterns.

Despite these limitations, this provides a novel and adaptable surveillance approach for environmentally driven diseases and demonstrates the role of landscapes in driving malaria infection and detection. Incorporation of forest data enables identification of cost-effective risk-based surveillance approaches which increase probabilities of detecting malaria infections and can be applied to support elimination efforts. Additionally, the process-based modelling method used provides a flexible framework to quantify detection probabilities of different surveillance approaches.

## Data Availability

The datasets presented in this article are not readily available because these require approvals from relevant ethics committees. Requests to access the datasets should be directed to kfornace@nus.edu.sg.

## References

[1] Fornace KM Diaz AV Lines J Drakeley CJ. Achieving global malaria eradication in changing landscapes. Malar J. (2021) 20:69. doi: 10.1186/s12936-021-03599-0, 33530995 PMC7856737

[2] De Castro MC Monte-Mor RL Sawyer DO Singer BH. Malaria risk on the Amazon frontier. Proc Natl Acad Sci USA. (2006) 103:2452–7. doi: 10.1073/pnas.0510576103, 16461902 PMC1413719

[3] World Health Organisation. Malaria surveillance, monitoring and evaluation: A reference manual. Geneva: World Health Organization (2018).

[4] Okell LC Bousema T Griffin JT Ouedraogo AL Ghani AC Drakeley CJ. Factors determining the occurrence of submicroscopic malaria infections and their relevance for control. Nat Commun. (2012) 3:1237. doi: 10.1038/ncomms2241, 23212366 PMC3535331

[5] Okell LC Ghani AC Lyons E Drakeley CJ. Submicroscopic infection in plasmodium falciparum-endemic populations: a systematic review and meta-analysis. J Infect Dis. (2009) 200:1509–17. doi: 10.1086/644781, 19848588

[6] Hsiang MS Ntshalintshali N Kang Dufour MS Dlamini N Nhlabathi N Vilakati S . Active case-finding for malaria: a three-year national evaluation of optimal approaches to detect infections and hotspots through reactive case detection in the low transmission setting of Eswatini. Clin Infect Dis. (2019) 1316–1325. doi: 10.1093/cid/ciz403 PMC7318780 31095677

[7] Zhou G Afrane YA Malla S Githeko AK Yan G. Active case surveillance, passive case surveillance and asymptomatic malaria parasite screening illustrate different age distribution, spatial clustering and seasonality in western Kenya. Malar J. (2015) 14:41. doi: 10.1186/s12936-015-0551-4, 25627802 PMC4318448

[8] Sesay SSS Giorgi E Diggle PJ Schellenberg D Lalloo DG Terlouw DJ. Surveillance in easy to access population subgroups as a tool for evaluating malaria control progress: a systematic review. PLoS One. (2017) 12:e0183330. doi: 10.1371/journal.pone.0183330, 28813522 PMC5558981

[9] Hobbs NT Hooten MB. Bayesian models: A statistical primer for ecologists. New Jersey, USA: Princeton University Press (2015).

[10] Nelli L Ferguson HM Matthiopoulos J. Achieving explanatory depth and spatial breadth in infectious disease modelling: integrating active and passive case surveillance. Stat Methods Med Res. (2020) 29:1273–87. doi: 10.1177/0962280219856380, 31213191

[11] DOH (2018). Malaria control program [online]. Department of Health, Philippines. Available online at: https://www.doh.gov.ph/malaria-control-program [Accessed 19 Aug 2025].

[12] Reyes RA Fornace KM Macalinao MLM Boncayao BL De La Fuente ES Sabanal HM . Enhanced health facility surveys to support malaria control and elimination across different transmission settings in the Philippines. Am J Trop Med Hyg. (2021) 104:968–78. doi: 10.4269/ajtmh.20-0814 33534761 PMC7941801

[13] Blanco NFD Salvacion AR Devanadera MCE Abucay ER Sandalo RA. Characterizing malaria spatial distribution in the province of Palawan, Philippines. Spat Inf Res. (2022) 30:279–89. doi: 10.1007/s41324-022-00429-6

[14] Macalinao MLM Fornace KM Reyes RA Hall T Bareng APN Adams JH . Analytical approaches for antimalarial antibody responses to confirm historical and recent malaria transmission: an example from the Philippines. Lancet Reg Health West Pac. (2023) 37:100792. doi: 10.1016/j.lanwpc.2023.100792, 37693871 PMC10485684

[15] Araza AB Castillo GB Buduan ED Hein L Herold M Reiche J . Intra-annual identification of local deforestation hotspots in the Philippines using earth observation products. Forests. (2021) 12:1008. doi: 10.3390/f12081008

[16] Hansen MC Potapov PV Moore R Hancher M Turubanova SA Tyukavina A . High-resolution global maps of 21st-century forest cover change. Science. (2013) 342:850–3. doi: 10.1126/science.1244693, 24233722

[17] Fornace KM Surendra H Abidin TR Reyes R Macalinao MLM Stresman G . Use of mobile technology-based participatory mapping approaches to geolocate health facility attendees for disease surveillance in low resource settings. Int J Health Geogr. (2018) 17:21. doi: 10.1186/s12942-018-0141-0, 29914506 PMC6006992

[18] Facebook Connectivity Lab, and Center for International Earth Science Information Network (Ciesin) Columbia University. High resolution settlement layer (HRSL). New York: CIESIN, Columbia University (2016).

[19] Philippine Statistics Authority (2016). "Philippine population density (based on the 2015 census of population)"

[20] Weiss DJ Nelson A Gibson HS Temperley W Peedell S Lieber A . A global map of travel time to cities to assess inequalities in accessibility in 2015. Nature. (2018) 553:333–6. doi: 10.1038/nature25181, 29320477

[21] Lloyd CT Sorichetta A Tatem AJ. High resolution global gridded data for use in population studies. Sci Data. (2017) 4:170001. doi: 10.1038/sdata.2017.1, 28140386 PMC5283062

[22] Fick SE Hijmans RJ. Worldclim 2: new 1-km spatial resolution climate surfaces for global land areas. Int J Climatol. (2017) 37:4302–15. doi: 10.1002/joc.5086

[23] Land Processes Distributed Active Archive Center (Lp Daac) (2015). "Advanced spaceborne thermal emission and reflection radiometer global digital elevation model (ASTER GDEM) version 2" Sioux Falls, South Dakota NASA EOSDIS land processes DAAC, USGS Earth Resources Observation and Science (EROS) Center, NASA, USA.

[24] Alos-2/Alos Science Project Earth Observation Research Center (Eorc). (2017) "Global PALSAR-2/PALSAR/JERS-1 mosaic and Forest/non-Forest map" Japan Aerospace Exploration Agency (JAXA), Japan.

[25] Hepler SA Kaufeld KA Benedict K Toda M Jackson BR Liu X . Integrating public health surveillance and environmental data to model presence of histoplasma in the United States. Epidemiology. (2022) 33:654–9. doi: 10.1097/EDE.0000000000001499, 35545229 PMC9345522

[26] Hepler SA Kaufeld KA Kline D Greene A Gorris ME. Estimating coccidioidomycosis endemicity while accounting for imperfect detection using spatio-temporal occupancy modeling. Am J Epidemiol. (2024) 194:56–63. doi: 10.1093/aje/kwae199 39013787

[27] Redding DW Tiedt S Lo Iacono G Bett B Jones KE. Spatial, seasonal and climatic predictive models of Rift Valley fever disease across Africa. Philos Trans R Soc Lond Ser B Biol Sci. (2017) 372:20160165. doi: 10.1098/rstb.2016.0165, 28584173 PMC5468690

[28] Rue H Martino S Chopin N. Approximate Bayesian inference for latent gaussian models by using integrated nested Laplace approximations. J Royal Stat Soc Series B. (2009) 71:319–92. doi: 10.1111/j.1467-9868.2008.00700.x

[29] Lindgren F Rue H. Bayesian spatial modelling with R-INLA. J Stat Softw. (2015) 63, 1–25. doi: 10.18637/jss.v063.i19

[30] Martins TG Simpson D Lindgren F Rue H. Bayesian computing with INLA: new features. Comput Stat Data Anal. (2013) 67:68–83. doi: 10.1016/j.csda.2013.04.014

[31] Dacuma MGB Dimalibot JC Baril JA Allian F Bahidjan DK Mori V . Subpatent plasmodium with mutant pfmdr1, pfcrt, and pvmdr1 alleles from endemic provinces in Mindanao, the Philippines: implications for local malaria elimination. Int J Infect Dis. (2021) 110:45–53. doi: 10.1016/j.ijid.2021.06.033, 34157387

[32] Seth MD Popkin-Hall ZR Madebe RA Budodo R Bakari C Lyimo BM . Prevalence of subpatent plasmodium falciparum infections in regions with varying transmission intensities and implications for malaria elimination in mainland Tanzania. Malar J. (2025) 24:101. doi: 10.1186/s12936-025-05341-6, 40140867 PMC11948789

[33] Slater HC Ross A Felger I Hofmann NE Robinson L Cook J . The temporal dynamics and infectiousness of subpatent plasmodium falciparum infections in relation to parasite density. Nat Commun. (2019) 10:1433. doi: 10.1038/s41467-019-09441-1, 30926893 PMC6440965

[34] Tobin RJ Harrison LE Tully MK Lubis IND Noviyanti R Anstey NM . Updating estimates of plasmodium knowlesi malaria risk in response to changing land use patterns across Southeast Asia. PLoS Negl Trop Dis. (2024) 18:e0011570. doi: 10.1371/journal.pntd.0011570, 38252650 PMC10833542

[35] Stark KD Regula G Hernandez J Knopf L Fuchs K Morris RS . Concepts for risk-based surveillance in the field of veterinary medicine and veterinary public health: review of current approaches. BMC Health Serv Res. (2006) 6:20. doi: 10.1186/1472-6963-6-20, 16507106 PMC1409776

[36] Hansen MC Krylov A Tyukavina A Potapov PV Turubanova S Zutta B . Humid tropical forest disturbance alerts using Landsat data. Environ Res Lett. (2016) 11:034008. doi: 10.1088/1748-9326/11/3/034008

[37] Bailey LL Mackenzie DI Nichols JD. Advances and applications of occupancy models. Methods Ecol Evol. (2013) 5:1269–79. doi: 10.1111/2041-210X.12100

