# Supplementary material for: Integrating forest data and health facility surveys to optimise risk-based malaria surveillance in the Philippines
Source: Front Public Health. 2025 Dec 18;13:1699392. doi: 10.3389/fpubh.2025.1699392 (PMC12756170; doi:10.3389/fpubh.2025.1699392)
Supplement: Supplementary file 1 [file Supplementary_file_1.docx]

**Supplementary Information: Integrating forest data and health facility surveys to optimise risk-based malaria surveillance in the Philippines**

*Study site:*

Surveys were conducted in 27 health facilities across Rizal municipality in Palawan, the Philippines (Figure S1). This municipality has a primarily rural population of approximately 50,000, including a high proportion of indigenous groups.

**Figure S1.** Estimated travel time to surveyed health facilities


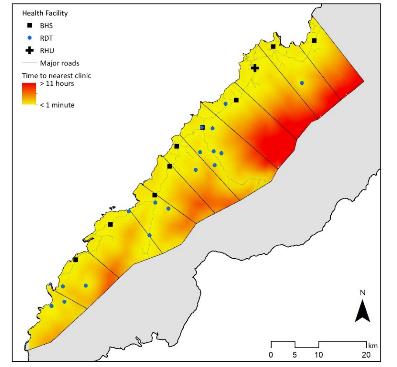


**Figure S2.** All identified household locations in Rizal with households sampled by A. enhanced surveillance and B. PCD
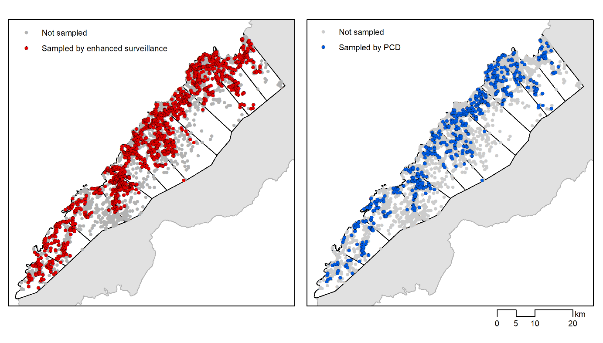


Using high resolution settlement layers, the 2015 geolocated census and self-reported residence locations from study participants, we identified 7313 unique residence locations at 50m resolution. Using these locations, we extracted plausible spatial and environmental covariates for malaria infection and treatment seeking behavior (Table S1). All data was resampled to 30m resolution and processed using R statistical programming language v3.6. Maps were visualized using R or ArcGIS (ESRI, Redlands, USA). For forest data obtained from (1), we classified forest as 50% canopy cover and added forest gain layers while subtracting annual forest loss layers to obtain forest cover for the year.

**Figure S3.** Overview of occupancy modelling approach to assess detection based on survey and diagnostic methods


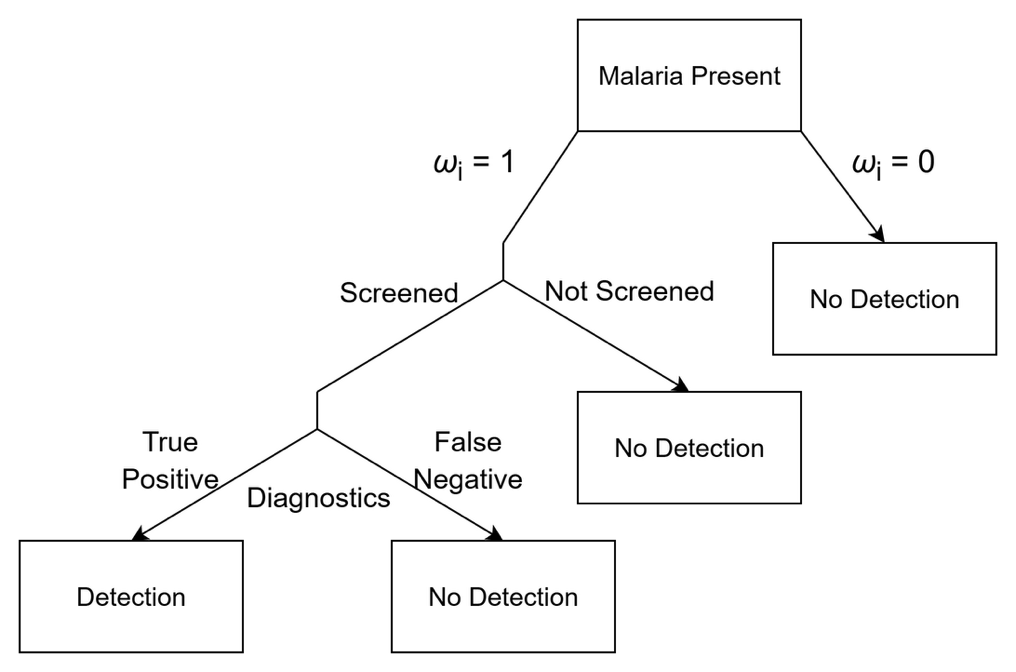


**Table S1.** Spatial and environmental data

| **Covariate** | **Description** | **Spatial Resolution** | **Source** |
| --- | --- | --- | --- |
| Elevation | Metres above sea level (MSL) | 30m | ASTER GDEM (2) |
| TWI | Topographic wetness index, log(m^2^/m) | 30m | Calculated from ASTER GDEM using dynamic TOPMODEL (2, 3) |
| Upslope area | Upslope area, log(m^2^/m) | 30m | Calculated from ASTER GDEM using dynamic TOPMODEL (2, 3) |
| Aspect | Compass direction slope is facing (degree) | 30m | ASTER GDEM (2) |
| Population density | 2015 population density, census adjusted | 100m | WorldPop (4) |
| Forest distance | Euclidean distance to forest in 2017 (m) | 30m | Calculated from (1) |
| Recent deforestation | Euclidean distance to forest loss in the past year (m) | 30m | Calculated from (1) |
| Historical deforestation | Euclidean distance to forest loss in the past 5 years (m) | 30m | Calculated from (1) |
| Forest/ non-Forest | Euclidean distance to forest classified as over 90% canopy cover with minimum area of 0.5ha | 25m | Calculated from 2016 PALSAR-2/ PALSAR Forest/Non-Forest Map derived from Synthetic Aperture Radar (SAR) (5) |
| Road distance | Euclidean distance to roads, calculated from GPS tracks | 10m | Calculated from field data |
| Travel time to clinic | Travel time to sampled clinics, calculated from friction surfaces | 30m | Calculated from (6) |
| Bioclimatic variables | Monthly averages of bioclimatic variables for 1970 - 2000 | 30s | Calculated from WorldClim version 2 (7) |

We summarised environmental and spatial characteristics of all residence locations within the municipality as well as for households sampled under enhanced surveillance and standard PCD (Table S2).

**Table S2.** Baseline characteristics of all residence locations and locations sampled under different surveillance approaches

|  | **All residences** | **Enhanced Surveillance** | **Standard PCD** |
| --- | --- | --- | --- |
| Total number (n) | 7313 | 2201 | 698 |
| Travel time to closest health facility (minutes)* | 5.89 (3.18, 17.95) | 9.33 (3.64, 25.30) | 8.40 (3.40, 22.78) |
| Distance to main roads (m)* | 183.6 (30.0, 1218.2) | 466.2 (72.1, 1719.7) | 392.0 (71.1, 1544.2) |
| Population density (people per ha) * | 0.68 (0.47, 0.85) | 0.59 (0.38, 0.77) | 0.60 (0.40, 0.77) |
| Elevation (meters above sea level) * | 18.27 (12.01, 36.19) | 23.36 (13.34, 46.46) | 23.82 (13.70, 43.36) |
| Distance from forest (m) * | 27.50 (0.00, 61.49) | 27.59 (0.00, 55.00) | 27.50 (0.00, 55.00) |
| Distance from closed canopy forest (m)* | 534.1 (30.0, 1827.0) | 182.5 (0.00, 1081.7) | 174.9 (0.00, 1055.2) |
| Temperature annual range (°C) ** | 9.40 (9.23, 9.50) | 9.45 (9.31, 9.59) | 9.44 (9.31, 9.55) |
| Annual precipitation (mm)** | 1629 (1602, 1683) | 1647 (1614, 1690) | 1644 (1614, 1689) |
| Precipitation of wettest month (mm)** | 260.1 (257.4, 267.8) | 261.8 (258.9, 269.7) | 261.6 (258.6, 271.2) |

* Median, IQR

** calculated from WorldClim monthly averages for 30-year period

Models were fit separately for enhanced surveillance and standard PCD and assessed based improvements to Deviance Information Criteria (DIC) as described in Methods (Table S3).

*Occupancy modelling*

**Table S3.** Model fit statistics for A. standard PCD and B. enhanced surveillance

| **Model** | **DIC** | **Detection DIC** | **Infection DIC** | **Total DIC** |
| --- | --- | --- | --- | --- |
| **Standard: passive case surveillance with routine diagnostics** | | | | |
| M_PD0_: univariate model for detection | 4608.62 |  |  |  |
| M_PD1_: univariate model for detection + covariates | 4516.27 |  |  |  |
| M_PD2_: univariate model for detection + covariates + spatial effect | 4341.68 |  |  |  |
| M_PO0_: univariate model for infection | 1771.23 |  |  |  |
| M_PO1_: univariate model for infection + covariates | 1611.58 |  |  |  |
| M_PO2_: univariate model for infection + covariates + spatial effect | 1548.53 |  |  |  |
| M_PJ1_: joint model for detection and infection + covariates + detection spatial effect + infection spatial effect |  | 4340.76 | 1550.49 | 5891.25 |
| M_PJ2_: joint model for detection and infection + covariates + detection spatial effect + infection spatial effect + shared spatial random effect |  | 4323.25 | 1554.63 | 5877.88 |
| **Enhanced: health facility-based surveillance with routine and molecular diagnostics** | | | | |
| M_PD0_: univariate model for detection | 8948.46 |  |  |  |
| M_PD1_: univariate model for detection + covariates | 8382.95 |  |  |  |
| M_PD2_: univariate model for detection + covariates + spatial effect | 7572.20 |  |  |  |
| M_PO0_: univariate model for infection | 2265.01 |  |  |  |
| M_PO1_: univariate model for infection + covariates | 2076.11 |  |  |  |
| M_PO2_: univariate model for infection + covariates + spatial effect | 2028.85 |  |  |  |
| M_PJ1_: joint model for detection and infection + covariates + detection spatial effect + infection spatial effect |  | 7542.48 | 2030.29 | 9572.77 |
| M_PJ2_: joint model for detection and infection + covariates + detection spatial effect + infection spatial effect + shared spatial random effect |  | 7515.99 | 2032.74 | 9548.73 |

Separate models of all locations with malaria infections were used to estimate a location specific probability of patent malaria, equivalent to the sensitivity of RDT or microscropy (Figure S2).

**Figure S4.** Mean posterior probability of patent malaria


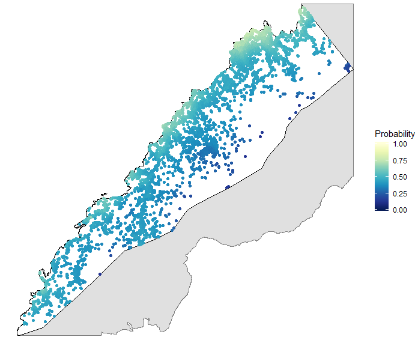


*Evaluation of surveillance systems*

Informed by these models we stratified households by distance from closed canopy forest, conducting sensitivity analysis to explore the impacts of using different thresholds of distance from forested areas (Figure S4).

**Figure S5.** Stratification of households by different thresholds of distance from closed canopy forests


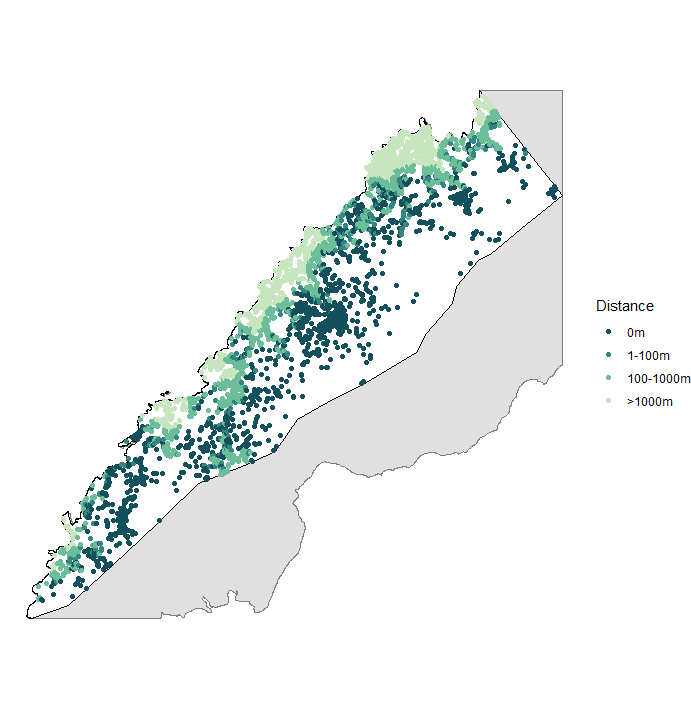


To evaluate surveillance approaches against the true underlying prevalence, we estimated the true presence of malaria infections from all available spatial information and molecular diagnostics, accounting for detection bias. The probability of surveillance approaches to detect infected locations was evaluated against these estimates of malaria presence using 10,000 posterior samples (Figure S4).

**Table S4.** Number of locations sampled and infections detected within risk zones defined by distance from forest

| **Area surveyed** | **Number of locations sampled** | **Number of infected locations detected** |
| --- | --- | --- |
| All Rizal | 2201 | 342 |
| Households within closed canopy forest | 764 | 199 |
| Households within 100m of closed canopy forests | 966 | 230 |
| Households within 1km of closed canopy forests | 1629 | 299 |

**Figure S6.** Posterior estimates of infections not detected under different surveillance scenarios (Table 3), including A. standard PCD, B. enhanced surveillance using health facility surveys and molecular diagnostics, C. PCD with molecular diagnostics, D. health facility surveys and routine diagnostics


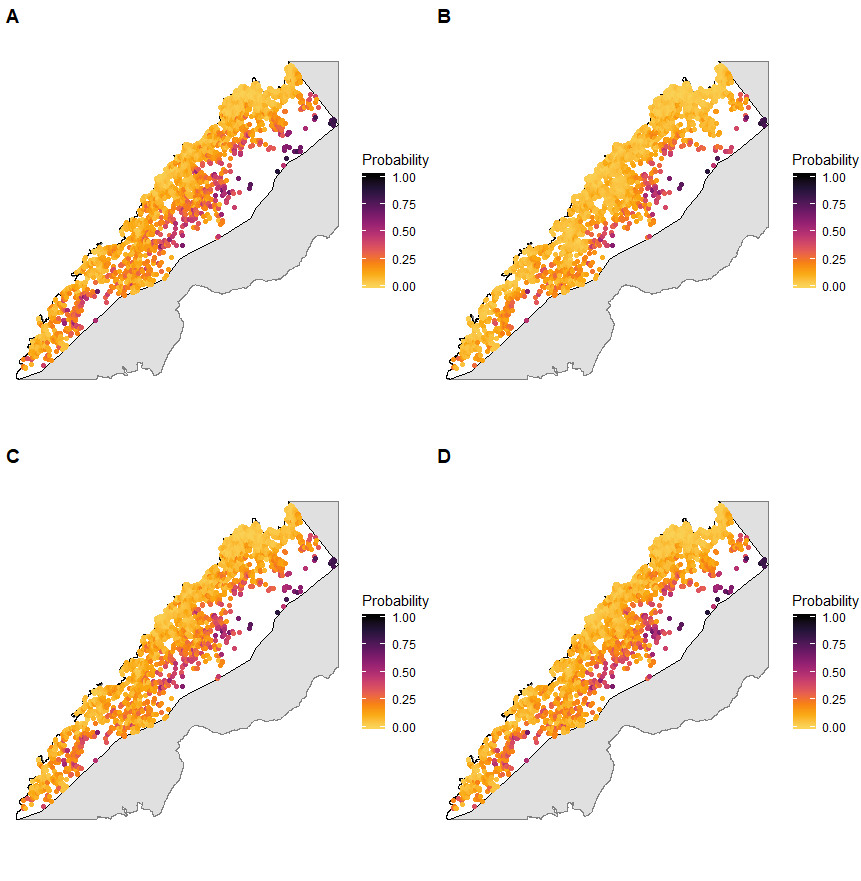


To evaluate cost effectiveness, we estimated additional costs for health facility surveys and diagnostics methods. This excluded capital costs and costs incurred by standard PCD under existing health surveillance systems. Costs were calculated in Philippine pesos (PHP) converted into US dollars (USD) using the 2018 conversion rates.

**Table S6.** Additional costs for health facility surveys and diagnostic methods for one year (excluding capital costs and existing health surveillance systems)

| **Category** | **Item** | **Quantity** | **Item Cost (PHP)** | **Total (PHP)** | **Total (USD)** |
| --- | --- | --- | --- | --- | --- |
| Personnel | RDT staff supplement | 17 | 12000 | 204000 | 4080 |
|  | BHS staff supplement | 9 | 18000 | 162000 | 3240 |
|  | RHU staff supplement | 3 | 18000 | 54000 | 1080 |
| Training | Annual RDT training costs per RDT centre | 17 | 3500 | 59500 | 1190 |
|  | Annual training costs per BHS Microscopist | 9 | 14375 | 129375 | 2588 |
| Equipment | RDT kit | 850 | 150 | 127500 | 2550 |
|  | Technician (molecular/ field) | 1 | 88000 | 88000 | 1760 |
|  | Lancets (5 boxes per station per year) | 135 | 350 | 47250 | 945 |
|  | Glass slides (10 boxes/ microscopist/ year) | 120 | 150 | 18000 | 360 |
|  | Alcohol (10 bottles/ microscopist/ year) | 120 | 350 | 42000 | 840 |
|  | Cotton (10 boxes/ microscopist/ year) | 120 | 85 | 10200 | 204 |
|  | Slide boxes (5 boxes/ microscopist/ year) | 60 | 300 | 18000 | 360 |
|  | Giemsa stain (1 bottle/ microscopist/ year) | 12 | 300 | 6000 | 120 |
|  | Immersion oil (1 bottle/ microscopist/ year) | 12 | 1200 | 14400 | 288 |
|  | Tablets | 27 | 6000 | 162000 | 3240 |
|  |  |  | **Total** | **1142225** | **22845** |

The costs of enhanced surveillance included the total additional costs to health facilities, including additional RDT and microscopy costs (Table S4). The costs of DNA extraction using Chelex and PCR for all samples was USD 25.20 (PHP 1260). We additionally included costs of using a commercial Qiagen DNeasy extraction kit (Qiagen, Hilden, Germany) and confirmatory PCR for 10% of samples at a cost of USD 44.00 (PHP 2200) per sample. The total cost of molecular tests per sample was USD 29.60; with 5767 individuals from 2201 locations included, the total cost of molecular diagnostics for enhanced surveillance was USD 170,703.20, with a cost per location included of USD 77.56. The costs of applying molecular diagnostics to standard PCD was calculated as the sum of the molecular costs for the 1914 febrile patients screened (USD 56,654.40). Estimates of the costs of health facility surveys included additional costs to health facilities (Table S4), excluding costs molecular diagnostics. Health facility survey costs were not scaled to reflect the number of individuals screened as these included staff salaries, training and purchase of standard quantities of supplies prior to the start of surveys. Similarly, estimates of the cost of risk-based surveillance were calculated as the sum of molecular costs for households included in this risk zone and the total costs of health facility surveys.

To determine how to define the high-risk zone for risk-based surveillance, we compared including all households within closed canopy forest areas to buffer distances of 100m and 1km. Based on the accuracy (as measured by households not detected) and costs of these definitions, we chose an intermediate risk stratification of all households within 100m of closed canopy forests (Figure S5).

**Figure S7.** Evaluation of definitions of high-risk zones for risk-based surveillance by distance from forest cover, including A. Estimated number of locations with malaria infections missed and B. Total cost per correctly identified location of malaria infection


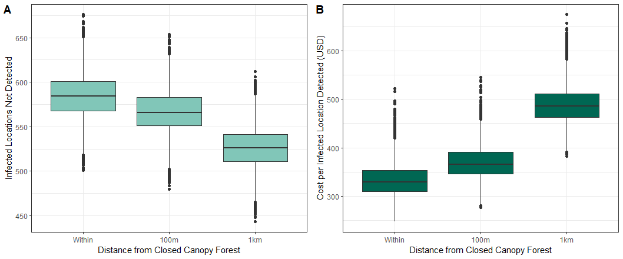


1. Hansen MC, Potapov PV, Moore R, Hancher M, Turubanova SA, Tyukavina A, et al. High-resolution global maps of 21st-century forest cover change. Science. 2013;342(6160):850-3.

2. Advanced Spaceborne Thermal Emission and Reflection Radiometer Global Digital Elevation Model (ASTER GDEM) Version 2 [Internet]. NASA EOSDIS Land Processes DAAC, USGS Earth Resources Observatoin and Science (EROS) Center. 2015. Available from: <http://gdem.ersdac.jspacesystems.or.jp/>.

3. Metcalfe P, Beven K, Freer J. Dynamic TOPMODEL: a new implementation in R and its sensitivity to time and space steps. Environmental Modelling and Software. 2015;72:155-72.

4. Lloyd CT, Sorichetta A, Tatem AJ. High resolution global gridded data for use in population studies. Sci Data. 2017;4:170001.

5. Global PALSAR-2/PALSAR/JERS-1 Mosaic and Forest/Non-Forest map [Internet]. Japan Aerospace Exploration Agency (JAXA). 2017. Available from: <http://www.eorc.jaxa.jp/ALOS/en/palsar_fnf/fnf_index.htm>.

6. Weiss DJ, Nelson A, Gibson HS, Temperley W, Peedell S, Lieber A, et al. A global map of travel time to cities to assess inequalities in accessibility in 2015. Nature. 2018;553(7688):333-6.

7. Fick SE, Hijmans RJ. Worldclim 2: New 1-km spatial resolution climate surfaces for global land areas. International Journal of Climatology. 2017.
